# Twice-Weekly 36-Hour Intermittent Fasting Practice Attenuates Hunger, Quadruples ß-Hydroxybutyrate, and Maintains Weight Loss: A Case Report

**DOI:** 10.7759/cureus.57979

**Published:** 2024-04-10

**Authors:** Katarina T Borer

**Affiliations:** 1 Diabetes and Endocrinology, School of Kinesiology, University of Michigan, Ann Arbor, USA

**Keywords:** daily caloric restriction, beta-hydroxybutyrate, modified intermittent fasting, sleep quality, energy expenditure, fat loss, weight loss, uninterrupted fast, appetite, intermittent fasting

## Abstract

Intermittent fasting (IF) approach to weight loss obviates the inconvenience of calorie counting required in daily caloric restriction (DCR). A metabolic defense mechanism (MDM) obstructs weight loss and facilitates weight regain possibly by increasing hunger and efficiency of exercise energy expenditure (EEf), and by reducing resting metabolic rate (RMR) and energy expenditure (EE) including physical activity (PA). IF may test whether its paradigm can better counteract MDM than DCR. A knowledge gap exists about whether the duration of weekly uninterrupted fasts (UFs), when the IF protocols are isocaloric, affects the MDM. The aim and objective of this 82-week study were to determine whether 36 hours of near-absolute twice-weekly UF will exacerbate MDM but generate similar rates of weight and fat losses compared to four IF studies featuring 20 hours of weekly UF with both IF protocols matched for weekly hours of fast (108) and free access to food (60), a fasting-to-eating (F/E) ratio of 1.8. This case report presents results of twice-weekly fasting on non-consecutive days (5:2-NC) and compares them to results from a 4:3-NC protocol with a 20-hour UF caused by a modification of providing a 500-600 kcal meal on three fasting days (M4:3-NC). Because the large meal raises insulin concentration for four hours at the start of the fasting day, the 20-hour UF consists of the remaining eight hours on the fasting day, followed by 12 additional nocturnal hours of fasting. The hypotheses were that (1) because of their matched F/E ratio, the rates of weight and fat losses will be similar in both protocols, and (2) because of its longer UF period, hunger will be higher and RMR and EE will be lower, in 5:2-NC than in M4:3-NC protocol. The main findings were that the 5:2-NC protocol produced (1) slower rates of weight and fat losses, (2) modest reduction in the sensation of hunger and substantial decline in fullness, (3) no change in RMR and EE, and (4) fourfold post-fast increase in the circulating concentration of the ketone body ß-hydroxybutyrate (BHB), 2.5 greater than in the M4:3-NC protocol. The absence of increased hunger and changes in EE, the variability of the rate of weight loss in the 5:2-NC protocol, plus increased EEf in one M4:3-NC study, suggest that IF does not mitigate MDM, but that shortened UF period in M4:3-NC reduces the rise in BHB. Thus, the addition of a large meal on fasting days is unnecessary for the prevention of hunger and is counterproductive for increases in BHB and its potential health benefits. Continuous practice of the 5:2-NC protocol allows sustained weight loss and maintenance of lost weight with diminished hunger for as long as it is implemented.

## Introduction

Since 1975, human overweight and obesity have increased exponentially to become a global health problem [1] driving efforts toward reduction of excess weight. The non-pharmacological and non-surgical approaches to weight loss have long utilized conventional daily caloric restriction (DCR). Its serious drawbacks were a usual failure to retain the weight loss over the longer term [2,3] and the monotony of daily dietary restriction coupled with the inconvenience of calorie counting. The extent to which a resistance to body mass loss, and its regain, reflect the operation of a body-mass protecting metabolic defense mechanism (MDM) is not fully understood. Manifestations of MDM include increased hunger and drive to eat during energy restriction or body mass loss [4,5], reduced motivation for energy expenditure (EE) of physical activity (PA), and reduction in resting metabolic rate (RMR) and lean body mass (LBM) [6]. Additional MDM effects are increased energy efficiency of exercise (EEf) [7], and increased insulin sensitivity driving fat re-synthesis and storage [8]. A new view of MDM is that energy deprivation preserves total energy expenditure (TEE) by making muscle contractions more efficient [7] and by suppressing energy-costly processes of weight and fat gain, immune defenses, reproduction, growth, and stress responses [9,10].

Within the past decade, intermittent fasting (IF) has raised interest in whether it may produce more sustainable weight loss without increased hunger or the inconvenience of calorie counting. A number of different IF protocols were developed [11], ranging from eating and fasting on alternate days to fasting on two (protocol 5:2-NC) or three (protocol 4:3-NC) nonconsecutive days per week. Based on Pub Med Commons tally, interest in, and publication of, IF studies have increased exponentially between 2002 and 2023.

Two distinguishing features of different IF protocols are the ratio of hours of fasting relative to hours of eating (the fasting-to-eating (F/E) ratio) and the duration of uninterrupted hours of fasting per unit of time. IF and DCR protocols with high F/E ratios predominantly influence weight loss. Currently, it is not clear whether longer uninterrupted fast (UF) durations would potentiate MDM by increasing hunger, reducing TEE including PA, and increasing the EEf of movement. This is of particular interest in view of the paucity of IF protocols with longer UF durations due to the prevalent modification of adding a large 500- to 600-kcal breakfast on fasting days. A 500 kcal meal increases plasma insulin concentration for about four hours and raises its area under the curve (AUC) to about 296 μIU/ml*min [12]. A 100 kcal meal increases plasma insulin for only three hours and reduces its AUC by 45% (134 μIU/ml*min). If the 100 kcal of food are distributed into three daily meals, each insulin excursion would last about one hour, and its AUC would be expected to be 15% as large as that of the 500 kcal meal (45 μIU/ml*min). IF protocols with longer UF periods would reduce the prevalence of the sustained high-insulin postprandial state conducive to energy storage and weight and fat gain that characterizes the feeding pattern prevailing in the USA. In the general population, the distribution of meals and snacking was reported to take place over 19 circadian hours with only five-hour daily UF periods [13].

The benefit of longer UF periods involves switching to a longer fasting metabolic state that relies on the mobilization of stored fat and the use of lipids for energy. This stimulates the production of the ketone body ß-hydroxybutyrate (BHB) which serves as an alternate fuel for the brain, heart, skeletal muscle, and kidney when plasma glucose declines [14]. BHB concentration gradually increases from <0.1 mM/L under the ad-libitum feeding state to a peak of 7 mM/L after 25 days of starvation [15]. BHB has powerful signaling functions [16]. It epigenetically activates gene networks that turn on lipid metabolism and enzymes that promote energy mobilization and utilization and mitochondrial biogenesis, while suppressing biosynthetic pathways. BHB also elicits sirtuin 1 release [17] a regulator of the aging process [14], and increases stress resistance while suppressing inflammation [18].

The present case report provides the opportunity to assess the effect of the 82-week-long practice of near-absolute 36-hour UF on two nonconsecutive days per week on the weight loss pattern, manifestations of MDM, and changes in appetite, EE, and BHB concentration. The outcome of this 5:2-NC protocol is compared to four studies that implemented the M4:3-NC protocol and reported on some of the same variables that were measured in the case report. All four M4:3-NC studies were also selected for differing in the 20-hour long UF duration and for the F/E ratio of 1:8 that was isocaloric to the 5:2-NC protocol.

The hypotheses for these comparisons are that (1) due to equal F/E ratios, 5:2-NC and M4:3-NC protocols and isocaloric DCR controls will exhibit comparable rates of weight, fat, and LBM losses; (2) due to its longer 36-hour UF duration, the 5:2- NC IF protocol will affect MDM manifestations by increasing hunger and EEf, and by decreasing RMR, EE, and levels of voluntary PA.

This article was previously posted to the Research Square preprint server on December 3, 2023, under the title "Effects of duration of uninterrupted fast in weekly intermittent fasting: Comparison of an 82-week 5:2 case report to an isocaloric modified 4:3 protocol under RSID: rs-3701752." This article is not pending full publication elsewhere.

## Case presentation

Materials and methods for the IF 5:2-NC protocol

Subject

A single female subject carried out a self-directed IF 5:2-NC protocol over 574 days or 82 weeks. The subject’s age was 82 years, and body mass, fat mass, and LBM were 70.9, 29.9, and 52.2 kg, respectively, at the start. BMI was 25.4 kg/m^2^. Blood pressure was 116.2 systolic, and 65.1 mmHg diastolic.

Dietary Intake

A Mediterranean dietary pattern was selected for its established health value and followed on the non-fasting days. A review [19] defining this diet indicated that it contained three to nine servings of vegetables, half to two servings of fruit, one to three servings of cereals, and up to eight servings of olive oil daily. The diet on four eating days closely matched the proposed foods. Breakfasts included baked oatmeal with apples and sour cream or kefir, nuts (almonds, hazelnuts, walnuts), pancakes with berries, and cottage cheese crepes sweetened with Stevia. On one day, it was a vegetable omelet. The beverage was coffee with milk or tea. Lunch was Swiss cheese or canned tuna fish on toast with avocado, onion, and tomato, or homemade bone or bean soups. Dinners included two cups of mixed salad with olive oil and vinaigrette dressing, brown rice, a green vegetable, and chicken or fish (salmon, cod, tilapia). Apples, grapes, pineapple, mango, nuts, occasional dark chocolate, cookies, or home-baked pies were eaten for dessert. The beverage was tea or coffee with milk. Meals were eaten at 07:00, 12:00, and 16:30 hours. On Tuesday and Friday fasting days, the total energy allotment was 87 kcal to 97 kcal. It consisted of a 110-112 g slice of cantaloupe (about 40 to 50 kcal) and three hazelnuts (9 kcal/1.25 g nut, 85% fat, each) divided into three meals. The breakfast beverage was 125 ml of low-sugar orange juice containing 5 g of sugar (20 kcal), while green tea or water was consumed at other times.

Anthropometric Measures

The measurement procedures typically implemented in energy restriction studies [20] were followed. Body weight was measured daily at 07:00 hours on a physician’s mechanical beam scale (Ava Weigh MSB440 lb). Body fat and fat-free or LBM were measured on a bioimpedance scale (Tanita Wb-100a, Tanita Corporation, Tokyo, Japan) eight times, at the start, and on days 1, 185, 257, 345, 412, 450, 505, and 574. Height was measured intermittently with a stadiometer in the health center. Fasting plasma glucose, hemoglobin A1c, triglycerides, and low-density and high-density cholesterol were measured by the university hospital laboratory during week 1 and week 82.

Appetite Assessments

Appetite ratings utilized the 100-cm visual analog scale (VAS) method [21]. Meals were restricted to a 9.5-hour eating window between 07:00 and 16:30 hours. Hunger and appetite on fasting days and three to four non-fasting days were assessed daily between days 225 and 251 at the following times: 07:00, 09:00, 11:00, 13:00, 15:00, 17:00, and 19:00 hours. They also were reported as AUCs.

Blood Pressure

A validated Omron Evolv wireless upper-arm blood pressure monitor (www.omronhealthcare.com) [22] measured upper-arm blood pressure once a month at 07:00 hours.

Energy Expenditure Measures

PA, total daily EE, heart rate, and sleep phases and quality were measured daily with a Fitbit Versa 2 activity tracker and its software (www.fitbit.com) accessible on the cell phone daily and from the Fitbit weekly email reports. This device has been used in medical publications for measurements of heart rate [23], EE [24], and sleep studies [25]. The device tracked the daily number of steps and distance traveled. Besides daily walking, the subject engaged in two hours per week of Ashtanga yoga and resistance exercise using weight machines in the university gym. RMR was measured with the Tanita bioimpedance apparatus on days 1, 185, 257, 345, 412, 450, 505, and 574.

Sleep Phase Measurements and Sleep Quality

Daily records of minutes of sleep collected by the Versa-2 Fitbit activity monitor assessed durations of sleep phases. The variables tallied in nightly minutes were total, light (stages N1 and N2), deep (stage N3), rapid-eye-movement (REM) sleep, awake periods during the sleep period, as well as the number of brief awakenings. Sleep efficiency was estimated from the ratio of hours of wakefulness to hours of sleep [26].

Beta-Hydroxybutyrate

Beta-Hydroxybutyrate (BHB) was measured in lanced blood drops applied to ketone strips with the validated Precision Xtra blood glucose and ketone monitoring system (Abbott Diabetes Care Inc., Alameda, United States) which was found to produce reliable blood ketone measurements [27]. Measurements were done on days 251 through 265 between 7:00 and 7:30 hours after the 36-hour fast day and on the three post-fast eating days, D1, D2, and D3. A single measurement was done on day 266 after 60 hours of UF resulting from two consecutive days of fasting.

Statistics

In accordance with time series analysis which applies to repeated measurements of a single unit (in this case measurements generated by a single subject), descriptive statistics were used for all measurements of individual variables. GraphPad Prism software (https://www.graphpad.com/scientific-software/prism), version 9.5.0 (730)) was used for plotting linear variables and Locally Weighted Scatterplot Smoothing (LOESS) procedure (https://peltiertech.com/loess-smoothing-in-excel) for non-linear ones. Comparisons between the data from a single subject in this case report and the data from the subjects in four M4:3-NC studies were discussed categorically in terms of the presence or absence of expected manifestations.

Results for the 5:2-NC protocol

Body Weight Changes

**Figure 1:**
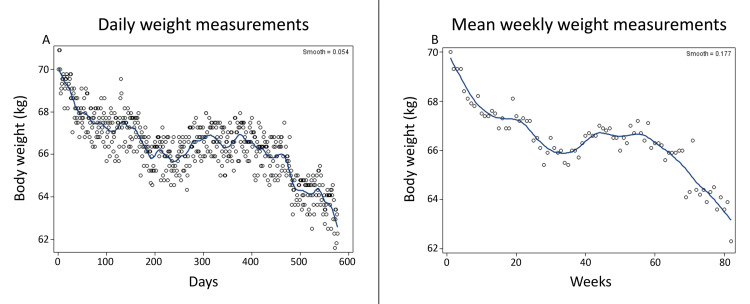
Daily (A) and mean weekly (B) weight changes Kg: Kilogram of body weight

Daily body weight changes are shown in Figure 1A and mean weekly changes are shown in Figure 1B. Weight loss was sustained through the 82 weeks of IF. Total weight lost in 82 weeks was 8.6 kg (0.11 kg/week) or 12.1% of starting weight. The LOESS plot of weekly weight changes in Figure 1B shows that the rate of weight loss during 82 weeks was variable (R^2^ = 0.58 for daily loss rate, R^2^ = 0.67 for mean weekly loss rate). There were three periods of rapid loss, the first 10 weeks (-0.25 kg/w), weeks 20 to 30 (-0.09 kg/w), and weeks 58 to 80 (-0.19 kg/w). In between, the rate of weight loss was slower between weeks 10 and 20 (-0.01 kg/w), unchanged between weeks 30 and 37 and 44 and 58, and weight even increased between weeks 38 and 44 (+0.25 kg/w).

Changes in Fat Mass, LBM, and BMI

**Figure 2:**
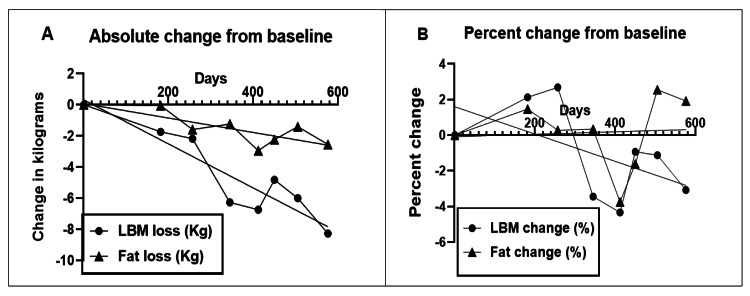
Absolute (A) and percent fat and lean body mass (LBM) changes Kg: Kilogram of body weight; LBM: Lean body mass

Absolute changes in body fat and LBM are shown in Figure 2A and percent changes in Figure 2B. Total fat mass lost in 82 weeks was 2.5 kg or 0.03 kg/w. The percent fat mass lost over 82 weeks was 11.8 or 0.14/w. Their linear regressions and coefficients of variance were y = -0.004641*x+0.08149, R^2^ = 0.91 (Figure 2A) and y = 0.0006954*x-0.08714, R^2^ = 0.92 (Figure 2B), respectively. Total LBM loss was 6.8 ± 0.1 kg or 0.08 kg/w and its linear regression was y = -0.01416*x+0.3287, R^2^ = 0.92. Percent LBM lost was 13.5 or 0.16/w and its linear regression was y = -0.01416*x+0.3287, R^2^ = 0.41.

**Figure 3:**
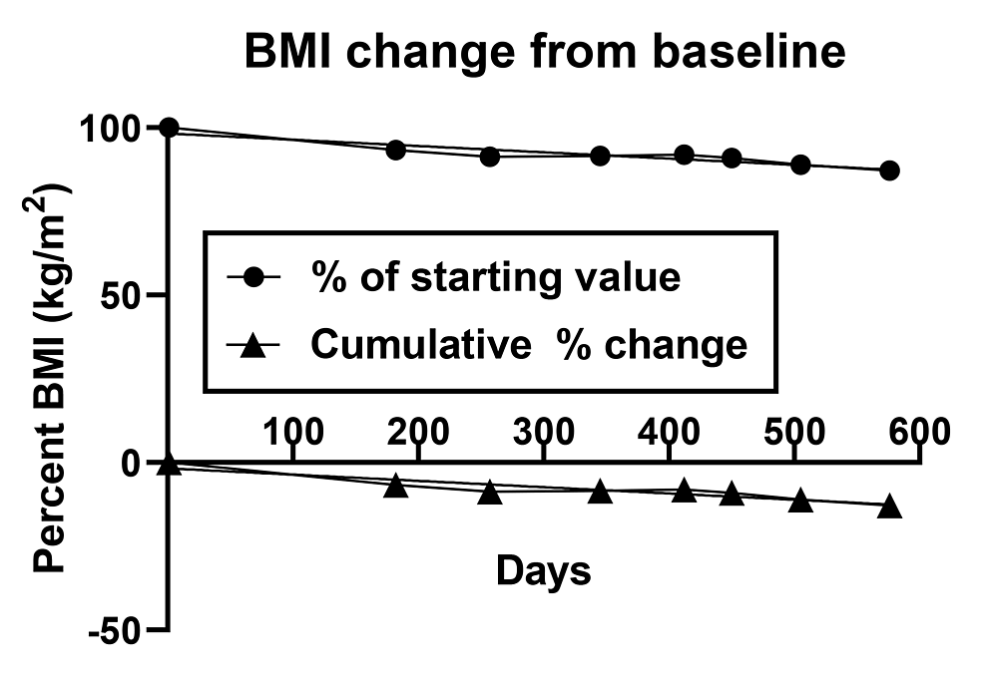
Absolute (A) and percent (B) body mass index (BMI) changes BMI: Body mass index (kilogram of weight divides by square of body height, kg/m^2^)

BMI change is shown in Figure 3. The absolute BMI decline over 82 weeks was 3.2 kg/m^2^ or -0.04/w. Percent BMI decline was 12.6% or 0.15%/w. Their regressions were y = -0.0048*x -0.4368, R^2^ = 0.87 and y = -0.0188*x -1.708, R^2^ = 0.87, respectively.

Blood Parameter Changes

 

Four of five parameters measured in, or derived from, plasma showed beneficial changes (Table 1). By week 82, fasting plasma glucose declined to 97 mg/dl, HbA1c to 5.6%, LDL-cholesterol to 69 mg/dl (0.6%/w), and triglycerides to 42 mg/dl (0.8%/w). There was no change in HDL-cholesterol (53 mg/dl).

**Table 1:** Effects of IF on measured variables in two study protocols AUC: Area under the curve for the VAS appetite ratings; BP: Blood pressure; BHB: ß-hydroxybutyrate; BMI: Body mass index (kg/m^2^); EE: Total daily energy expenditure; EEf: Efficiency of muscle contraction; HDL: High-density lipoprotein; Hg: Mercury; LBM: Lean body mass or fat-free mass; LDL: Low-density lipoprotein; NC: No change; NM: Not measured; NR: Not reported; REM: Rapid eye-movement sleep; RMR: Resting metabolic rate; RMRLBM: resting metabolic rate normalized by LBM; RQ: Respiratory quotient; TG: Plasma triglycerides; VAS: Visual analog scale for appetite ratings; VCO_2_: Rate of carbon dioxide production; VO_2_: Rate of oxygen consumption; >: Change to

Protocol 5:2-NC				Protocol M 4:3-NC		
Variable	Start	Week 82	Change	Study	IF change	DCR change
Age (years)	82	83	1	[22-25]	45.3 ± 1.3	
Body mass (kg)	70.9	63.3	-0.11/w	[22-25]	93.3 ± 1.2	91.5 ± 0.7
Weight loss (%)		12.2	0.15/w	[22-25]	-0.8 ± 0.05/w	-0.8 ± 0.1/w
Fat mass (kg)	21.2	18.7	-0.03/w	[22-25]	39.1 ± 2.8	39.7 ± 2.0
Fat mass loss (%)		11.8	0.14/w	[22-25]	-1.0 ± 0.4/w	-0.9 ± 0.4/w
Height (m)	1.67	1.67	0		NR	NR
LBM mass (kg)	50.5	43.6	-0.08/w	[22-25]	55.1 ± 1.6	53.6 ± 1.6
LBM loss (%)		13.5	0.16/w	[22-25]	-0.3 ± 0.1/w	-0.2 ± 0.1/w
BMI (kg/m^2^)	25.4	22.2	-0.04/w	[22-25]	32.9 ± 0.4	
BMI loss (%)		12.6	0.15/w	[23]	-0.75/w	-0.77/w
Fasting glucose (mg/dl)	99	97	-0.02/w	[24]	88.2 > 81.7	88.2 > 86.0
Hemoglobin A1c (%)	5.8	5.6	-0.002/w		NM	NM
LDL-cholesterol (mg/dl)	131	69	-0.76/w	[24]	112 > 97.8	116 > 110.2
HDL-cholesterol (mg/dl)	53	53	0	[24]	54.1 > 50.2	54.1 > 54
TG (mg/dl)	66	42	-0.3/w	[24]	106.2 > 84.1	115.1 > 101.8
Hunger AUC/day (%)	0	22.5		[25]	NC	NC
Fullness AUC/day (%)	100	30				
Food intake kcal/day	NM	NM		[23]	1,854 > 913	1,908 > 1,210
BP systolic (mmHg)	116.2	126.2	0.13/w	[23]	119 > 110.1	123 > 114.2
BP diastolic (mmHg)	65.1	69.1	0.05/w	[23]	70	74
EE (kcal/day)	1,598	1,587	-0.13/w		NR	NR
EEf (% ch in kcal to five-fold increase in effort)	NM	NM		[22]	0	93
							Steps walked /w	35,510	35,874	4.43/w	[23]	52,688	47,688
Distance walked (km/w)	24.6	24.4	-0.002/w		NR	NR
RMR (kcal/day)	1,226	1,206	-0.24/w	[22]	1,488 > 1,368	1,342 > 1,302
RMRLBM (kcal/day)	24.5	27.7	0.04/w	[22]	24.7 > 23.9	24.9 > 25
RQ (VCO_2_/VO_2_)	NM	NM		[22]	0.86 > 0.81	0.87 > 0.82
Total sleep (min/night)	414	474	0.7/w	[25]	NC	NC
Light sleep (min/night)	333	340	0.09/w			
REM sleep (min/night)	46.8	65	0.2/w			
Deep sleep (min/night)	46.8	67	0.2/w			
Sleep efficiency (hours of awake/sleep)	13.9	13.8	0.01/w		NR	NR
BHB (mM/L) fed > fast		0.14 > 0.66		[24]		0.03 > 0.26

Appetite Changes

**Figure 4:**
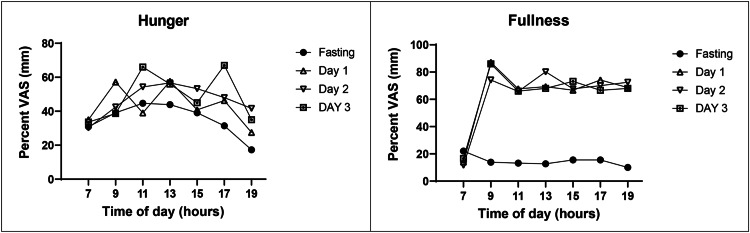
Changes in the VAS ratings of hunger (left panel) and fullness (right panel) measured at two-hour intervals between 07:00 and 19:00 hours VAS: Visual analog scale of appetite sensations

The VAS estimates of hunger and fullness were measured 11 times between weeks 32 and 36 at two-hour intervals between 07:00 and 19:00 hours during the fasting day and three post-fast days (Figure 4). The first-morning hunger rating on a VAS scale was similarly low on fasting and the three post-fast days (32.5 ± 1.2%) but rose to a peak between 11h and 15h (Figure 4). The average mid-day hunger rating was highest on post-fast D2 (45% higher than at 7 h) compared to the fasting day (38% higher) and post-fast days D1 (28% higher) and D3 (33% higher). The last daily hunger rating was lowest on the fasting day (-44% below 7h), became gradually attenuated on D1 (-21%) and D2 (- 8%), and on D3 was 24% higher than at 7 h.

The first-morning VAS fullness rating was similarly low on fasting and on the three post-fast days (16.2 ± 2.2%). After the onset of eating, and for the next five rating times, the fullness rating rose to 72 ± 0.6% and declined on the fasting day to 14.1%. During the last daily rating at 19 hours, fullness declined on fasting day by about 50% to 10% and by less than 3% on the three post-fast feeding days to 70%.

**Figure 5:**
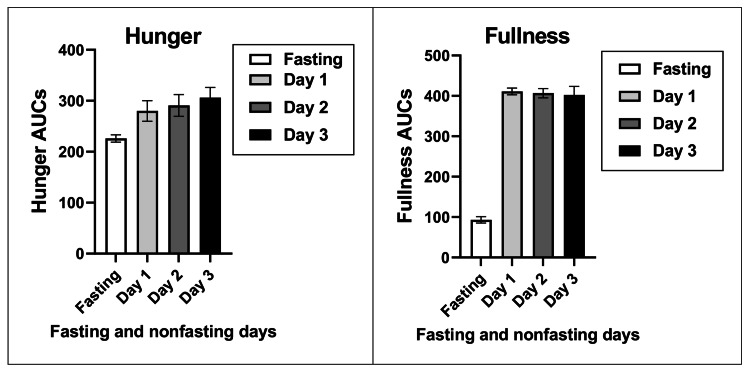
Areas under the curve for hunger (left panel) and fullness (right panel) on the fasting day and three subsequent days with ad-libitum access to food AUC: Area under the curve; D1, D2, D3: Appetite AUCs on three non-fasting days after the fasting day; Fasting: Appetite AUC during the fasting day

Both hunger and fullness AUC ratings differed between the fasting and the three post-fast days. Hunger rating AUCs during the fasting day were 19.3%, 22.1%, and 26.1% lower than on the post-fast ad-libitum eating days, D1 through D3. Fullness ratings were 77.3, 77.1, and 76.8 lower during the fasting day than on the three post-fast days (Figure 5).

Blood Pressure

**Figure 6:**
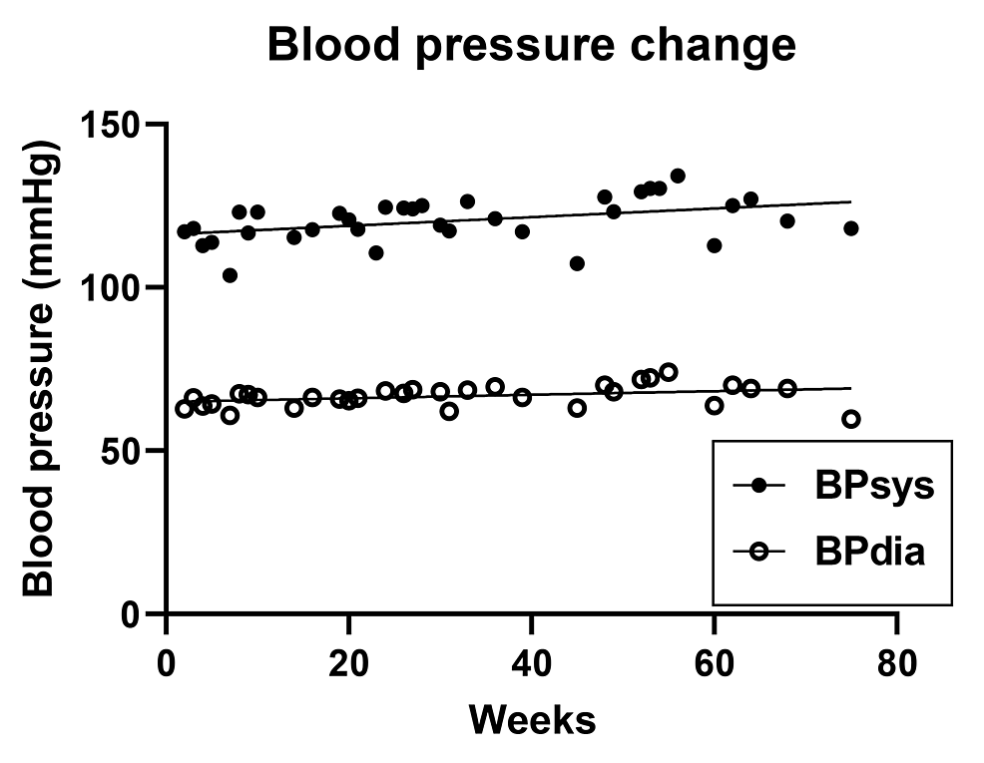
Time course of changes in systolic and diastolic blood pressure measurements BPdia: Diastolic blood pressure; BPsys: Systolic blood pressure; mmHg: Millimeters of mercury

Total increases in BPsys (y=0.1832*x + 114.97, R^2^=0.22) and BPdia (y=0.096*x +64.11, R^2^=0.295) were 10 and 4 mm Hg, respectively. BP increases were unexpected based on the usually negative correlation with body fat [28]. The average systolic (BPsys) and diastolic (BPdia) blood pressure during 76 weeks of measurements were 125.5 ± 1.18 and 66.7 ± 0.6 mmHg, respectively (Figure 6).

Changes in the Measures of Energy Expenditure

**Figure 7:**
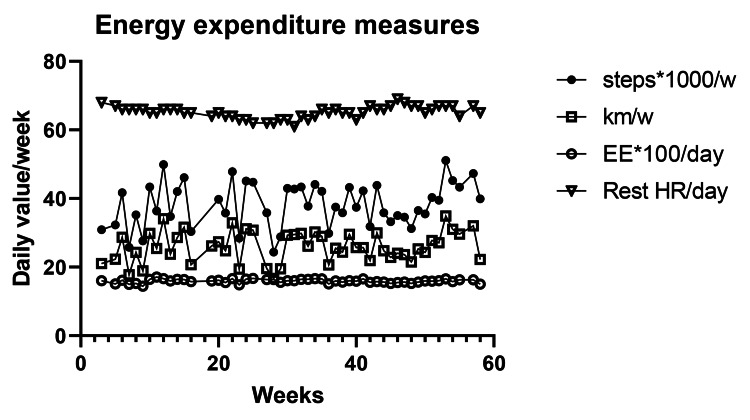
Energy expenditure measurements EE: Energy expenditure in kilocalories; Km: Kilometers walked; Rest HR/day: Resting heart rate in beats per day; w: Week

None of the four measures of EE changed significantly over 82 weeks of the study (Figure 7). Mean weekly measures for steps (35,873.6 ± 742.8) and kilometers walked (24.4 ± 0.5), total energy expended (1587.3 ± 6.5), and resting heartbeats during sleep (64.9 ± 0.2) changed over 82 weeks by a total of only 2.8 steps, 1.1 km, 0 kcal, and 0.02 heartbeats, respectively. None of their respective linear regressions (steps: y = 4.974*x+36931, R^2^ = 0.002, kilometers: 0.01357*x+25.13, R^2^ = 0.0007, kilocalories: -0.1626*x+1597, R^2^ = 0.0025, and heartbeats: 0.0094*x+64.78, R2= 0.0058) showed a substantial change during 82 weeks.

**Figure 8:**
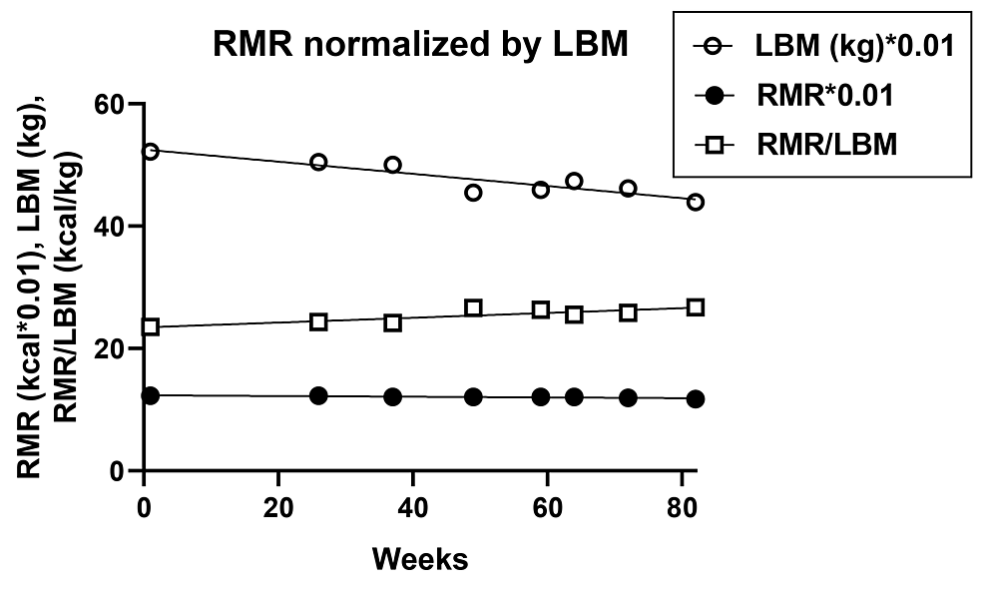
Changes in resting metabolic rate (RMR), lean body mass (LBM), and their ratio RMR: Resting metabolic rate; LBM: Lean body mass; RMR/LBM: Resting metabolic rate normalized per unit lean body mass

The starting RMR was 1,226 kcal and declined during 82 weeks by only 20 kcal. Although absolute LBM declined, RMR change, normalized by LBM to 24.5 ± 0.5 kcal/kg, increased by only 0.04 kcal/kg*week over 82 weeks (Figure 8).

Sleep Changes

**Figure 9:**
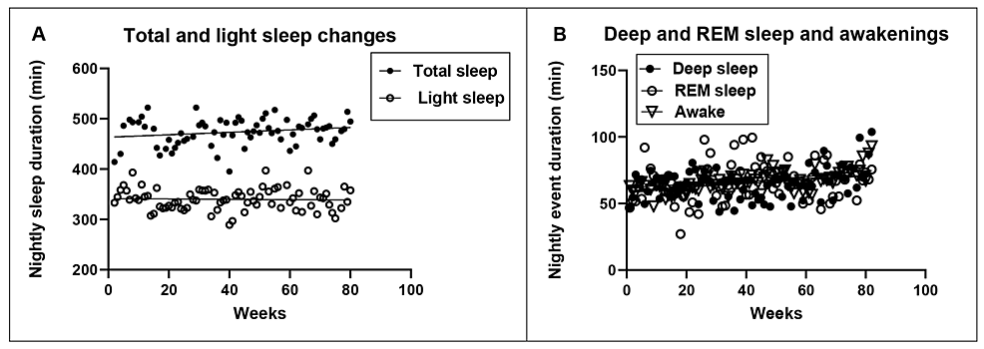
Changes in total nightly minutes of total and light sleep (A), deep and REM sleep and wakefulness during sleep period (B) REM: Rapid eye-movement sleep

Different measures of sleep are shown in Figure 9A (total and light sleep) and Figure 9B (deep and REM sleep and duration of wakeful minutes during the sleep period). Mean durations of total and light sleep were 473.8 ± 3.1 minutes or 7.9 hours, 340 ± 2.5 minutes or 5.7 hours. For deep and REM sleep, and wakefulness during the sleep period, durations were, respectively, 64.6 ± 1.4, 66.7 ± 1.7, and 66 ± 0.9 minutes or 1.1 hours. The percentages of light, deep, REM sleep, and the period of wakefulness as a function of total sleep duration were 71.8, 13.6, 14.1, and 13.9, respectively. The mean number of brief awakening episodes during the sleep period was 4.5 ± 0.1, or one percent of the total minutes of sleep. The slopes of linear regressions changed during 82 weeks of measurements only for deep sleep, wakeful periods (Figure 9B), and episodes of brief awakenings, but not for total, light, or REM sleep. Over 82 weeks, total sleep and light sleep decreased by a total of 19.2 and 2.3 minutes, and deep and REM sleep and duration of wakefulness increased by 8.6, 8.9, and 15.9 minutes, respectively. Nightly awakenings increased by three minutes. Average sleep efficiency, estimated as hours of wakefulness divided by hours of sleep [26] was 13.9%, with a decrease of 0.01 efficiency units per week.

ß-hydroxybutyrate Changes

**Figure 10:**
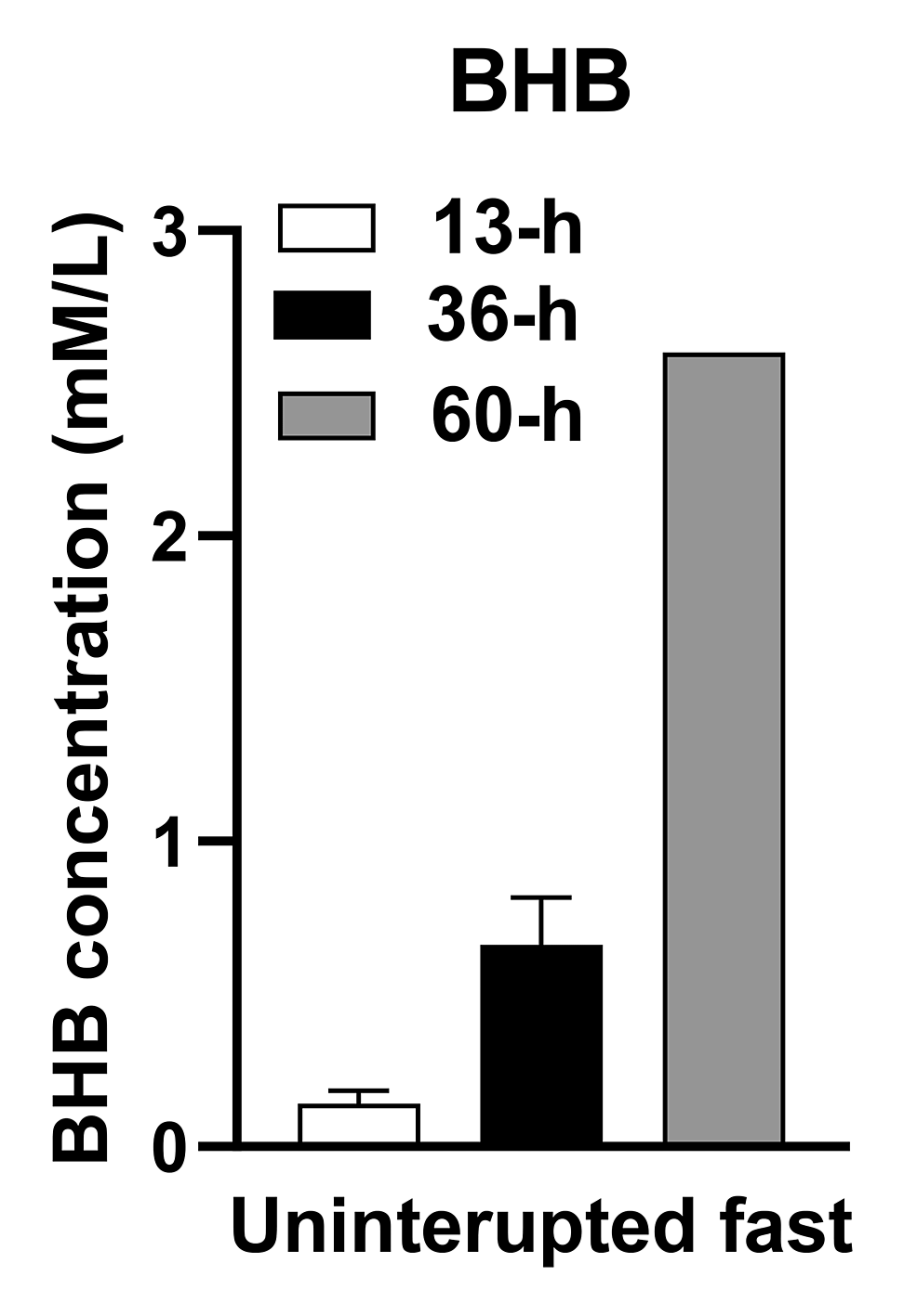
Beta-hydroxybutyrate (BHB) concentrations as a function of duration of uninterrupted fast BHB: ß-hydroxybutyrate, a ketone body; H: Hour; L: Liter; mM: Millimoles

Figure 10 shows the mean BHB concentration in the morning after a 12-h overnight UF, after 36 hours of near-absolute fast on non-consecutive days, and after 60 hours of near-absolute fast on two consecutive days. BHB concentration after 36 hours of near-absolute fast was 0.66 ± 0.07 mM/L, 4.4 times higher than the 0.14 ± 0.02 mM/L measured after the 12-hour overnight fast on non-fasting days. A 60-h UF following two consecutive days of eating fewer than 100 kcal/day, produced a BHB concentration of 2.6mM/L, 17.3 times higher than on free-feeding days.

Discussion of the 5:2-NC-IF protocol results

Eighty-two weeks of near-absolute 36-hour fasts on two non-consecutive days per week revealed substantial changes in several anthropometric, blood pressure, and BHB measurements, variable changes for components of appetite and sleep, and no change in the measures of EE.

Anthropometric Measures

Weight loss was sustained over 82 weeks at an average rate of -0.1 kg/w (Table 1), but it was not linear (Figure 1B). During the first ten weeks, it was at its fastest (-0.25 kg/w), but there were periods of slower loss, weight stabilization, and even weight gain. The rate of fat mass loss was more consistent, on average at -0.11 kg/w or -0.03 kg/w (Figure 2A), but its percentage loss was only 0.14/w (Figure 2B). LBM also declined at -0.08 kg/w or -0.16%/w (Figure 2B). The rate of decline in BMI was 0.04 kg/m^2^ or 0.15% per week (Figure 3). All measures of plasma chemistry displayed various degrees of improvement, particularly the LDL-cholesterol and plasma triglycerides (Table 1).

Appetite

There is considerable interest in the extent to which IF influences appetite. A number of studies reported increased hunger after prolonged DCR fasts [4,5]. Higher post-fast hunger is considered one of the components of MDM that drives increases in food intake, obstructs weight loss, and promotes weight regain. VAS measurements, carried out between weeks 32 and 36, the period of lowest midpoint weight level, revealed a small decrease in hunger ratings on fasting days relative to non-fasting days, and a very strong and consistent suppression of fullness ratings (Figure *4*). This contrast was also evident in the comparison of hunger and fullness AUCs (Figure* 5*) where hunger AUCs were 22.5% lower on fasting days than on feeding days, while fullness AUCs were 77% lower. These data suggest first, that declines in the sensation of fullness are greater and better detected than those of hunger, and second, that the 5:2-NC IF protocol characterized by 36 hours of near-absolute fast twice a week does not produce a significant increase in hunger after a 5.2 kg body weight loss during the period appetite was examined.

Energy Expenditure

Measures of EE over 82 weeks of 5:2-NC IF were consistently stable. No significant changes were detected in either daily calories expended, number of weekly steps, and kilometers walked (Table 1), or resting heart rate. RMR declined by only 20 kcal over 82 weeks, an effect that changed to a slight increase after normalization for LBM change. It, therefore, appears that sustained slow weight loss using this IF protocol produces losses in body fat, LBM, and RMR that are proportional to losses in total body mass, while the measures of EE remain stable. Our data are, therefore, supportive of the constrained model of energy regulation which posits that EE is regulated and protected at the expense of body mass and fat losses, fertility, growth, and immune and stress defenses as reported by others [9,10]. Our data also do not support the hypothesis that this IF protocol suppresses the operation of MDM as suggested by great variability in the rate of weight loss that was at times suppressed or even reversed.

Sleep Variables

Sleep parameters were stable and within the healthy absolute ranges. Although the slopes of the linear regressions of deep sleep, nocturnal wakefulness, and brief awakenings, were significant, the clinical relevance of these changes to overall health is questionable because of the small magnitude of absolute changes (8.6, 15.9, and 3-minute increases over 82 weeks, respectively). There was no change in sleep efficiency over 82 weeks of sleep study.

BHB Concentrations

A very clear and significant change was a 4.4-fold rise in BHB concentration in the mornings after 36 hours of near-absolute fast compared to the value achieved after a 12-hour fast. Given that BHB concentration asymptotes at 7 mM/L after 25 hours of starvation [15], the rise in BHB concentration from 0.14 mM/L after a 12-hour fast to 0.66 mM/L after a 36-hour fast, and to 2.6 mM after a 60-hour fast, clearly shows a strong positive association between the concentration of BHB and the duration of near-absolute UF (Figure 10).

Conclusions for protocol 5:2-NC results

Self-directed twice weekly near-absolute 36-hour fast can be conducted relatively comfortably and effectively over an extended period of one and a half years. It produces a sustained, but modest and variable, rate of body mass and fat losses, a slight decrease in hunger with a greater decrease in fullness, modest increases in blood pressure, no changes in measures of EE or sleep phases and efficiency, and 4.4-fold increase in the concentration of BHB. Reduced body mass can be sustained for as long as the 5:3-NC protocol is practiced.

Materials and methods for the M4:3-NC protocol

Four modified IF studies [29-32] were selected for their measuring some key variables common to the protocol 5:2-NC, for having the same F/E ratio of 1:8, and for having a 44% shorter UF of 20 hours vs. 36-hours UF in 5:2-NC protocol. The mean age of an average of 20 IF subjects and their 14 DCR controls was 45.3 ± 1.3 years. The body mass, fat mass, and LBM were, for the IF subjects 93.3 ± 1.2, 43.9 ± 2.1, and 55.1 ± 1.6 kg, and for their DCR controls, 91.5 ± 0.7, 43.9 ± 1.5, and 53.6 ± 1.6 kg, respectively. The mean BMI for both groups was 32.9 ± 0.4 kg/m^2^ (Table 1). Two studies lasted 12 weeks [29,30] and the other two, using only women subjects [31,32], lasted eight weeks. The equivalence in the percentage of dietary restriction between IF and control groups was defined in only one study as being 33% [29]. In the study [30] caloric intakes for IF and control groups were, respectively, specified as 550 to 800 kcal on fasting days and as 1,200 to 1,600 kcal on free-eating days. Of the remaining two studies, only [31] provided dietary and anthropometric measurements, and [32] used these data to generate secondary analyses. Both of these studies had unmatched levels of dietary restriction of 37% for the IF group and 32% for the controls, because IF subjects consumed 144 kcal per day less than planned. Uneven matching of the levels of energy restriction between the IF and control groups did not detract from the useful information about the presence, magnitude, and direction of changes of select variables in the protocol M4:3-NC for the purpose of comparison to results of protocol 5:2-NC.

All four M4:3-NC studies provided anthropometric measurements while the others yielded individual comparison values. RMR, respiratory quotient (RQ), and EEf were reported in the study [29], as were BMI, food intake, BP, and the number of steps in [30], blood metabolites and BHB in [31], and appetite and sleep efficiency in the study [32]. EEf (net efficiency) in the study [29] was established by measuring energy expended during bicycle ergometry at three pedaling intensities 10 W, 25 W, and 50 W by IF and control subjects. RQ measure in IF and control subjects was obtained by measuring the ratio of the rate of carbon dioxide (VCO_2_) produced over the rate of captured respiratory oxygen consumed (VO_2_) using a gas apparatus during exercise. An RQ of 1 indicates exclusive carbohydrate oxidation, while an RQ of 0.7 indicates 100% lipid fuel utilization. Appetite and sleep efficiency in the study [32] were measured with a three-factor eating questionnaire [33] and the sleep with Pittsburgh sleep quality index [26].

Results of the M4:3-NC IF and DCR protocols

M4:3-NC section of Table 1 presents results from the four IF protocols and their DCR controls. Mean anthropometric measurements were available for IF and control subjects in all four studies. The percent weekly body mass loss was -0.8 ± 0.1/w for both IF and control groups per week. Other respective percentage weekly losses were -1.0 ± 0.4 and -0.9 ± 0.4 for fat mass, -0.75/w and -0.77 for BMI, and -0.3/w and -0.2/w for LBM. Changes in circulating metabolic factors (Table 1) were measured only in the study [31] where the IF subjects were subjected to greater dietary restriction than the controls. Therefore two-to three-fold greater declines in absolute values of plasma glucose, LDL-cholesterol, and HDL-cholesterol in IF subjects compared to their controls could have been influenced by the dietary restriction difference. A five-fold greater decline in plasma triglycerides in IF subjects than in their controls is more likely to be a consequence of IF exposure. No group difference in the three-factor appetite-rating questionnaire [33] was reported in the study [32]. A 7% to 7.5% decline in systolic blood pressure was about equal in the IF and control subjects in the study [30] where both groups sustained equivalent dietary restrictions. The RQ data indicated that both the IF and control groups shifted by 5.7% to 5.8% of their energy consumption toward lipids in the study [29]. In the same study, IF subjects experienced 2.7 times greater loss in absolute RMR rating than the controls, and eight times so when the RMR was normalized by LBM. There also was a difference in the EEf of muscle contractions when IF and control subjects pedaled bicycles at 10 W, 25 W, and 50 W power [29]. IF subjects increased their EE by 93% at 50W compared to 10 W, while controls showed no change in response to rising effort. While these data suggest that the IF protocol selectively increased the energy efficiency of muscle contractions compared to isocaloric DCR controls, this conclusion is contradicted by evidence in the study [7] where subjects, stabilized at 10% weight loss over a period of weeks, showed a significant increase in EEf of muscle contractions. No changes in sleep score [26] were recorded for IF and control subjects in the study [32]. Finally, BHB was measured only in the study [31] where the IF subjects were more severely energy-deprived than the controls. Consequently, BHB increased from 0.03 mM/L on fed day to 0.26 mM/L after the fasting day.

Conclusions for M4:3-NC protocol results

While only four studies were examined for the effect of an IF protocol with a 20-hour long UF and similar dietary restriction between IF and DCR control groups, their outcomes differed very little after 8 and 12 weeks. Percent weekly rates of weight loss were almost identical for body mass for IF and control subjects, and they were only slightly greater for fat mass, LBM, and BMI, most likely due to greater dietary restriction in IF relative to control groups registered in the study [31] and in its duplicate [32]. Similarly, two to three-fold greater declines in plasma glucose, LDL, and HDL cholesterol in IF subjects relative to controls in the study [31] are likely caused by the same difference in dietary restriction. While systolic pressure declined by about 7%, the change was equivalent in IF and control subjects in the study [32]. Appetite did not differ in IF from the control group in the study [32]. Four results stand out as likely to be specific for the IF protocol as they were not observed in DCR controls, although one of them is contradicted by a DCR study [7]. A decline in plasma triglycerides was five times greater in the IF compared to control subjects in [31], both the absolute RMR, and its normalization by LBM, declined more in IF than in control subjects [29], and BHB increased from 0.03 mEq/L to 0.26 mEq/L selectively in IF subjects after the fasting day [31]. A striking 93% increase in net efficiency of muscle contraction in IF subjects but not in isocaloric DCR controls [29] is contradicted by a DCR study [7] where subjects, stabilized to a 10% weight loss, displayed a significant increase in energy efficiency of exercise. Thus, both the IF protocol and DCR weight loss support increases in muscle contraction efficiency.

## Discussion

Before discussing the outcome differences between the four M4:3-NC studies and a single 5:2-NC case report, it is necessary to point out several limitations of such a comparison. Starting with study subjects, there were significant differences (Table 1) in age almost by a factor of 2 (45.3 ± 1.3 in M4:3-NC vs. 82 years in 5:2-NC protocol), and respectively, in starting body mass: 92.4 ± 1.0 vs. 70.9 kg, in fat mass: 39.4 ± 2.0 kg vs. 20.9 kg, in percent body fat: 43.9 ± 1.8 vs. 32.8, and in the duration of IF intervention: 8 and 12 weeks vs. 82 weeks. Other significant limitations are that, with the exception of body mass and body fat changes, most variables in M4:3-NC, eligible for comparisons to protocol 5:2-NC, were analyzed in very few or single studies. Two M4:3-NC studies [31] and [32] were based on a single group of IF subjects measured in the study [31], and their IF group was more severely energy deprived (37%) than their DCR controls (32%). In only two studies by Coutinho et al. [29] and Steger et al. [30], dietary restriction in IF70 and DCR70 controls was matched. Despite these limitations, the usefulness of the outcome comparisons is in revealing a pattern and suggesting the direction and magnitude of IF-induced changes as a function of a difference in the duration of UF in studies matched for the F/E ratio.

The first hypothesis that, due to equal F/E ratios, 5:2-N protocol, M4:3-NC protocols, and their isocaloric DCR controls will exhibit comparable rates of change in anthropometric variables including weight, fat, and LBM losses, was not supported. The weight loss studies are predicated on the assumption that the magnitude of weight loss will bear a linear relationship to the quantity of restricted calories, regardless of the subject’s starting fat mass and without the interference of an MDM. In the present study, the 5:2-NC protocol (value columns one to three) and M4:3-NC studies (last two columns in Table 1) were matched for weekly F/E ratio of 1.8 (60 hours of feeding to 108 hours of fasting per week) with the expectation of an equal rate of weight loss. In contrast to the expectation, weekly rates of body mass loss in the M4:3-NC protocol were about six times higher (-0.8 kg/w) than in the 5:2-NC protocol (-0.13 kg/w). Similarly, the percent weekly fat mass losses were between 8.2 and 9 times higher (-0.9 and -1%/w) in the M4:3NC than in 5:2-NC protocol (-0.11%/w). The equality between the IF70 and DCR70 rates of weight loss in the four M4:3-NC studies confirms the conclusion reached in a systematic review of IF studies [34] that IF appears to produce weight reduction effects that are equivalent to continuous energy restriction (DCR) but does not explain the difference in the rate of weight and fat losses between the two comparison IF protocols.

One possible explanation for the difference in the rate of body mass and fat losses between the M4:3-NC and 5:2-NC protocols may have to do with differences in the duration of the comparison studies. The four M4:3-NC studies lasted between 6 weeks and 12 weeks, while the 5:2 protocol extended over 82 weeks. As Figure 1B shows, the rate of weight loss was faster at -0.28 kg/w during the first 10 weeks of IF in the 5:2-NC study than during weeks 10 to 60, when it averaged 0.04 kg/w. There also were periods of no loss or even some weight gain. Most of the M4:3-NC studies did not report continuous weight or fat measurements, and they were only either 8 weeks or 12 weeks long, so it can be assumed that at least some of the difference in the body mass and fat loss rates between the two protocols are attributable to the faster rates of losses during the early stages of fasting.

The second possible explanation for the difference in the rate of weight loss between the two protocols may be related to the difference in the starting body fat mass of their subjects. The size of the fat depot at 39.4 kg was 32% larger in the M4:3-NC subjects than in the 5:2-NC protocol. Body fat measurements in the 5:2-NC study were less frequent, but the rate of fat loss during the first 26 weeks of IF was faster at -0.3 kg/w than during the next 38 weeks at 0.06 kg/w. Most weight loss strategies aim for the loss of body weight and body fat but for the preservation of LBM. It is of interest that weekly percent losses of the LBM were about equal not only between IF70 and DCR subjects in M4:3-NC studies (-0.3/w in IF and -0.2/w in controls) but also between the M4:3-NC and the 5:2-NC protocol (-0.2/w). It is also of interest that the magnitude of percent weekly LBM loss in the 5:2-NC study was of the same magnitude as the rates of weight (-0.13/w) and fat loss (-0.11/w) in the 5:2-NC protocol, while in the M4:3-NC protocol the losses of body mass (-0.8/w) and body fat (-0.9 to 1.0/w) were between eight to nine times greater than the losses of LBM. This, again, would support the speculation that the size of the starting body and fat masses influence, in part, the rates of their loss, but not the rate of LBM loss. This issue deserves additional study.

Weight and fat loss usually accompany changes in circulating metabolites and blood pressure. The starting values in these variables showed some differences between subjects in the two protocols. The fasting glucose was 12% lower, and LDL cholesterol 14.5% lower, in M4:3-NC than in 5:2-NC subjects, while plasma triglycerides were 62.1% times higher in this protocol than in the 5:2-NC study. In the comparison of blood metabolites both the starting differences between the protocols and the actual protocol effects need to be taken into account. The protocol effect in lowering fasting plasma glucose was substantially greater (-7.4%) in M4:3-NC than in the 5:2-NC protocol (-2%) despite its substantially lower starting concentration. The HbA1c, however, declined by 3.4% in the 5:2-NC protocol possibly due to the longer measurement duration of a small protocol effect. The protocol effect in lowering the plasma LDL-cholesterol concentration in the 5:2-NC study was substantially greater (-47.2%) than in the M4:3-NC (-12.7%) protocol in accordance with the respective initial concentration difference. Starting HDL-cholesterol basically did not substantially change in either IF protocol. Contrary to the changes in LDL-cholesterol, plasma triglycerides both displayed greater starting concentration difference (62.1% higher in M4:3-NC than 5:2-NC protocol) and strong protocol effects. The decline in concentration was greater in the 5:2-NC case report (-36.4%) than in the M4:3-NC protocol (-20.8%).

An additional variable, often linked to changes in anthropometric values, is the quality of sleep. No changes were observed in the quality of sleep in the M4:3-NC study [32]. A more detailed analysis of sleep patterns in the 5:2-NC protocol uncovered almost no changes in the duration of total (473.8 minutes), light (340 minutes), and REM sleep (64.6 minutes) with only -19.2 minutes, -2.3 minutes, and 8.6 minutes change over 82 weeks. Increases of 8.6 minutes, 15.9 minutes, and 3 minutes, respectively, for deep sleep (66.7 minutes), periods of wakefulness (66 minutes), and number of awakenings (4.5) over 82 weeks showed very small differences.

Starting systolic blood pressure was comparable in the two protocols at between 116 mmHg and 119 mmHg, but the diastolic pressure was 13% higher in M4:3-NC subjects than in the 5:2-NC subjects. The divergence in the BP changes in the two protocols, with both pressures decreasing in the M4:3-NC subjects, and increasing in the 5:2-NC study may be attributable both to the difference in starting level of body fatness (BMIs of 32.9 ± 0.4 vs. 25.4 kg/m^2^), respectively, and in subject age (44.6 ± 2.6 vs. 82.5 years). Blood pressure change was reported to be positively related to body weight and fat losses [28], while advancing age has been negatively related to blood pressure, more so in women compared to men [35]. In both protocols, blood pressure values before and after the IF exposure remained largely within the healthy clinical range, where the ideal systolic to diastolic BP is represented by 90/60 mmHg and the normal healthy range by 140/90 mmHg.

The second hypothesis posited that the longer 36-hour duration of UF in the 5:2-NC IF protocol will affect manifestations of MDM including increased hunger and muscle EEf, decreased RMR, TEE, and levels of PA. Data addressing this hypothesis were provided in the study [29] and several measurements in the protocol 5:2-NC. The [29] study defined MDM as a reduction in total EE, driven by both a decline in RMR, reduced spontaneous PA, and an increase in EEf. Additional contributors to MDM are increased sensations of hunger and drive to eat, and reduced sensation of fullness.

The 5:2-NC protocol provided more information on appetite and sleep variables while no difference was found in the appetite between the IF and isocaloric DCR protocols in the study [32], a conclusion that was also reached in a systematic comparison and meta-analysis of IF and DCR studies [36]. In a study [32], a composite score reflecting dietary restraint, disinhibition, and hunger components in a questionnaire [33] reported no significant difference between IF (1.1) and DCR70 subjects (0). Reduced spontaneous food intake in IF subjects was reported in two M4:3-NC studies [30,31]. Food intake was not measured in protocol 5:2-NC, but an examination of both hourly VAS sensations of hunger and fullness and their areas under the curve on fasting and eating days was performed. VAS assessment of appetite was fully characterized in the 5:2-NC case report as producing on fasting days slightly, but still substantially lower, VAS hunger ratings, and a very strong and consistent suppression of VAS fullness ratings. On fasting days, AUCs of hunger ratings were 19% to 26% lower than on feeding days, while fullness AUCs were about 77% lower. Thus, the negative evidence regarding increases in hunger both in protocol 5:2-NC and in studies [31] and [32] does not justify modifying the weekly IF protocols by addition of the 500 kcal to 600 kcal meals on fasting days with the expectation of increased hunger. The addition of a 500 kcal to 600 kcal meal on fasting days in modified weekly IF protocols is not only unnecessary but also detrimental, in that it curtails increases in BHB which rise in proportion to the duration of UF. In the study [31] that supported only 20 hours of UF because of the large meal on fasting days, the increase in BHB was only 0.26 mM/L in the morning after the fasting day. In protocol 5:2-NC which allowed a UF of 36 hours of near absolute fast, the increase in BHB at 0.66 mM/L was 2.5 times greater. In view of the powerful signaling functions [16] of BHB, it would be beneficial to explore the health benefits of weekly fasting IF protocols with longer UF periods. BHB was shown to epigenetically activate gene networks that turn on lipid metabolism, enzymes that promote energy mobilization and utilization, and mitochondrial biogenesis, while suppressing biosynthetic pathways, and eliciting sirtuin 1 release [17].

No effect of the M4:3-NC protocol was reported for sleep quality [32], and no change in sleep parameters and sleep efficiency was found in protocol 5:2-NC.

Protocol 5:2-NC provided evidence that there were no differences in the levels of voluntary PA. The number of weekly steps and kilometers walked remained relatively constant over 82 weeks. Adding this to the evidence that total EE and RMR remain constant in both protocols. And that the RQ is not selectively affected by IF [29] supports the concept that EE is regulated in conjunction with the maintenance of LBM despite the losses of body mass and body fat. This hypothesis posits [9,10] that to preserve TEE during energy restriction, MDM operates by reducing body mass and fat mass, making muscle contractions more efficient [7,29], and suppressing other energy-costly processes supporting immunity, reproduction, growth, and stress responses [9,10]. We find no evidence that IF reduces or mitigates this process.

## Conclusions

The 5:2-NC protocol, which allows 36 hours of near absolute UF twice a week, supported slower but sustained and variable rates of weight and fat losses, a slightly lower sensation of hunger but a substantial decline in fullness, and a four-fold increase in the concentration of the ketone body BHB. This BHB increase after a 36-hour near absolute fast was 2.5-fold higher than after the 20 hours of UF in the isocaloric and modified three-days-a-week fasting protocol, M4:3-NC. The self-directed 5:2-NC protocol was comfortably tolerated over 82 weeks. The addition of the large meal on the fasting day to modified weekly IF protocols in expectation of increased hunger is therefore not necessary and carries the disadvantage of reducing the rise in BHB, an important signaling molecule with a number of beneficial epigenetic benefits. IF protocols did not change the parameters of EE or counteract manifestations of an MDM and its interference with weight loss and promotion of weight regain. The self-directed 5:2-NC protocol was comfortably tolerated over 82 weeks. The significant benefit of the 5:2-NC IF protocol is that it allows weight loss, increases BHB concentration, and maintains lost body mass without an increase in hunger, so long as it continues to be practiced.
